# Impaired LPS Signaling in Macrophages Overexpressing the P2X7 C-Terminal Domain or Anti-P2X7 C-Terminal Domain Intrabody

**DOI:** 10.3390/ijms26031178

**Published:** 2025-01-29

**Authors:** Chisato Sakuma, Takato Takenouchi, Mitsuru Sato

**Affiliations:** 1 Silkworm Research Group, Division of Silk-Producing Insect Biotechnology, Institute of Agrobiological Sciences, National Agriculture and Food Research Organization, Tsukuba, Ibaraki 305-8634, Japan; sakumac590@affrc.go.jp; 2 Animal Model Development Group, Division of Biomaterial Sciences, Institute of Agrobiological Sciences, National Agriculture and Food Research Organization, Tsukuba, Ibaraki 305-8634, Japan; ttakenou@affrc.go.jp

**Keywords:** macrophage, P2X7, lipopolysaccharide, myeloid differentiation primary response gene 88, Toll-like receptor 4, innate immunity

## Abstract

The P2X7 receptor is involved in innate immune responses, with its intracellular C-terminal domain capable of interacting with signaling molecules to regulate immune cell activation; however, the mechanisms underlying the signaling complexes remain unclear. To elucidate the function of the P2X7 C-terminal domain, we established bone marrow-derived macrophage (BMDM) cell lines from transgenic (Tg) mice overexpressing the C-terminal domain of P2X7 or anti-P2X7 C-terminal domain single-chain variable fragment (scFv) intrabody. In contrast to wild-type mouse BMDMs, the Tg BMDMs showed impairment of inflammatory responses induced by lipopolysaccharide (LPS) stimulation, such as NF-κB activation and subsequent TNF-α, IL-1β, and IL-6 expression. Furthermore, P2X7 was specifically associated with myeloid differentiation primary response gene 88 (MyD88) in wild-type BMDMs; its specific interaction was strongly interfered with by overexpression of the P2X7 C-terminal domain or anti-P2X7 C-terminal domain scFv in Tg BMDMs. These observations strongly suggest that P2X7 may have pivotal roles in LPS signaling cascades and could modulate macrophage inflammatory responses through its C-terminal domain.

## 1. Introduction

P2X7 is a member of the P2X family of receptors, which utilize ATP as a ligand [1]. Activation of P2X7 by high extracellular ATP concentrations opens permeable cation channels for K^+^, Na^+^, and Ca^2+^, resulting in various cellular responses, including morphological changes; lipase, kinase, and transcription factor activation; cytokine release; and apoptosis [2,3,4]. Unlike other members of the P2X family, P2X7 has a unique, long intracellular domain at the C-terminus, containing multiple protein- and lipid-interaction motifs, including Src homology 3 (SH3)-binding, TNFR1 death, cysteine-rich, and LPS-binding domains [5,6,7]. P2X7 is primarily expressed in immune cells, including macrophages, monocytes, and dendritic and microglial cells, and is involved in inflammatory responses [3,8].

In response to various pathogen-associated molecular patterns (PAMPs) and damage-associated molecular patterns (DAMPs), including extracellular ATP, NOD-, LRR and pyrin domain-containing protein 3 (NLRP3) undergo self-oligomerization to recruit adaptor apoptosis-associated speck-like protein containing a caspase-recruitment domain (ASC), which follows the recruitment of pro-caspase-1 and the formation of NLRP3 inflammasome. Assembly of the inflammasome induces caspase-1 self-cleavage and activation to cleave IL-1β and IL-18 to their mature forms [9,10]. The close interaction between P2X7 and NLRP3 was previously demonstrated, suggesting that P2X7 is involved in the activation of NLRP3 [11]. However, the precise binding site of P2X7 that interacts with NLRP3 remains unclear.

Hosts recognize PAMPs produced by invading microorganisms via pattern-recognition receptors. Toll-like receptors (TLRs), some of the most important pattern-recognition receptors, recognize various PAMPs and are central to the innate immune response against pathogens. LPS is a key component of the outer membrane of gram-negative bacteria and a ligand for TLR4. Following TLR4 activation by LPS, TIR domain-containing adaptor protein (TIRAP)/myeloid differentiation primary response gene 88 (MyD88) adapter-like (MAL) and TRIF-related adapter molecule (TRAM) act as bridging adaptors recruiting MyD88 and Toll/IL-1R domain-containing adaptor-inducing IFN-β (TRIF) to TLR4, respectively [12,13,14,15]. MyD88 recruits interleukin-1 receptor-associated kinase (IRAK) 1 and IRAK4, followed by IRAK4 phosphorylating IRAK1 [16]. Activated IRAK1 recruits the E3 ubiquitin ligase TNF receptor-associated factor 6 (TRAF6), which activates TGF-β-activated kinase 1 (TAK1), stimulating IKK-mediated NF-κB activation, leading to inflammatory cytokine expression [17,18,19].

Recently, P2X7 has been proposed to play important roles in LPS signaling through its C-terminal domain; however, the molecular mechanisms underlying the regulation of LPS responses by P2X7 remain unknown [20,21]. Here, we investigated the functions of the P2X7 C-terminal domain in LPS-induced macrophage activation by establishing bone marrow-derived macrophage (BMDM) cell lines from transgenic (Tg) mice overexpressing the C-terminal domain of P2X7 or single-chain variable fragment (scFv) intracellular antibody (intrabody), which specifically binds to the P2X7 C-terminal domain. This study aimed to uncover the role of P2X7 in the LPS signaling of macrophages through its C-terminal domain.

## 2. Results

### 2.1. Establishment of BMDM Cell Lines from Wild-Type, P2X7-CT2 Tg, and Anti-P2X7-scFv Tg Mice

**Figure 1:**
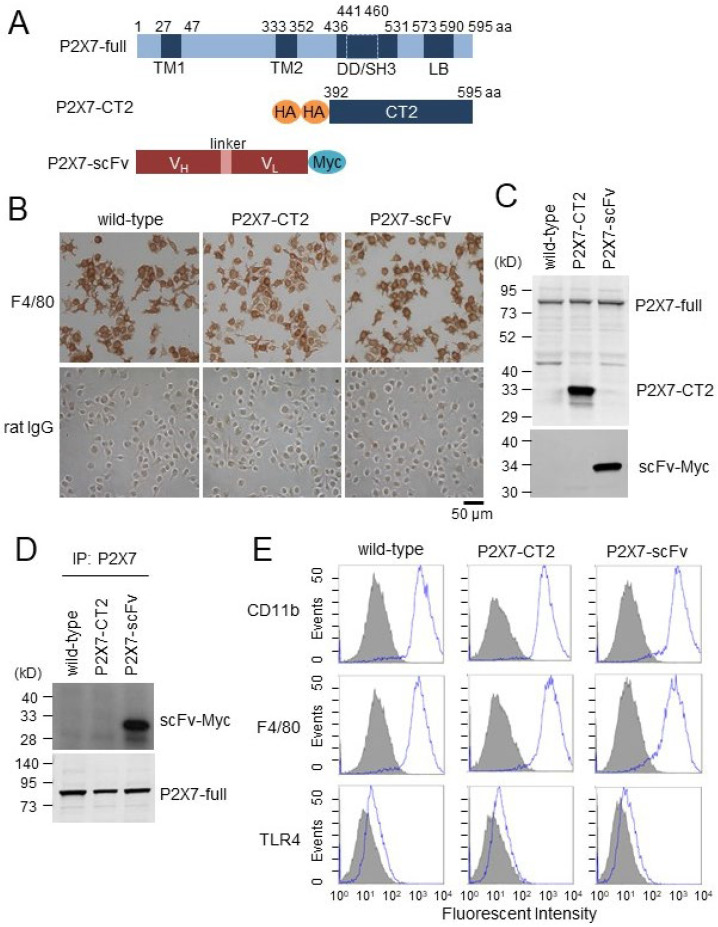
Characterization of bone marrow-derived macrophage (BMDM) cell lines from wild-type, P2X7-CT2 Tg, and anti-P2X7-scFv Tg mice. (**A**) P2X7, P2X7-CT2, and anti-P2X7-scFv are shown schematically. The major functional domains in P2X7: transmembrane (TM1, 27-47 and TM2, 333–352), TNFR1 death domain (DD, 436–531), Src homology 3 (SH3, 441–460), LPS binding (LB, 573–590). (**B**) BMDM cell lines were positively immunostained with anti-F4/80 antibody but not with control rat IgG. Bar = 50 µm. (**C**) Expression of truncated P2X7 (P2X7-CT2) and anti-P2X7-scFv in BMDMs were analyzed by Western blotting with an anti-HA tag antibody and anti-Myc tag antibody. (**D**) Wild-type, P2X7-CT2 Tg, and anti-P2X7-scFv Tg BMDMs were lysed and immunoprecipitated with anti-Myc tag antibody. Immunocomplexes and cell lysates were analyzed by Western blotting with an anti-P2X7 antibody. (**E**) Expression levels of cell surface molecules in wild-type, P2X7-CT2 Tg, and anti-P2X7-scFv Tg BMDM cell lines were investigated by FACS analysis with PE-conjugated anti-CD11b, anti-F4/80, and anti-TLR4 antibodies (blue line, open histogram) or isotype-matched control antibodies (histogram filled with gray). Full-length gels/blots and control data for immunoprecipitation and Western blot are presented in Supplementary Figure S1. All results are representative of three independent experiments.

We established Tg mice overexpressing the P2X7 C-terminal domain (P2X7-CT2) or anti-P2X7-scFv, specifically targeting the P2X7 C-terminal domain to inhibit P2X7 C-terminal domain function. P2X7-CT2 and anti-P2X7-scFv Tg mice were viable and fertile, with normal appearance under maintenance in a specific pathogen-free environment. Schematic representations of P2X7-CT2 and anti-P2X7-scFv are shown in Figure 1A. BMDMs prepared from wild-type, P2X7-CT2 Tg, and anti-P2X7-scFv Tg mice were immortalized by transducing with a c-myc gene-containing retroviral vector, and representative BMDM clonal cell lines were established. Wild-type, P2X7-CT2 Tg, and anti-P2X7-scFv Tg BMDM cell lines were strongly immunostained with the antibody against F4/80, a macrophage marker (Figure 1B). In contrast, these cell lines were not immunostained with control antibody rat IgG. Morphological and immunohistochemical features showed that these immortalized cell lines were derived from BMDMs.

Western blot analysis demonstrated sufficient expression of the truncated P2X7 (P2X7-CT2) and anti-P2X7-scFv in each of the P2X7-CT2 Tg and anti-P2X7-scFv Tg BMDMs (Figure 1C). In addition, immunoprecipitation analysis showed that anti-P2X7-scFv strongly binds to endogenous P2X7 in anti-P2X7-scFv Tg BMDMs (Figure 1D). Endogenous P2X7 was equivalently expressed in all BMDMs (Figure 1C,D).

To compare TLR4 expression levels among wild-type, P2X7-CT2 Tg, and anti-P2X7-scFv Tg BMDMs, we performed FACS analysis using anti-TLR4 antibody. TLR4 expression among BMDMs did not significantly differ. Furthermore, the expression levels of CD11b and F4/80 were also comparable among BMDMs (Figure 1E). These results suggest that the P2X7-CT2 and anti-P2X7-scFv expression does not influence the expression of the macrophage cell surface molecules CD11b, F4/80, and TLR4 in BMDMs.

### 2.2. LPS or Pam3CSK4-Induced Activation of NF-κB in BMDMs

**Figure 2:**
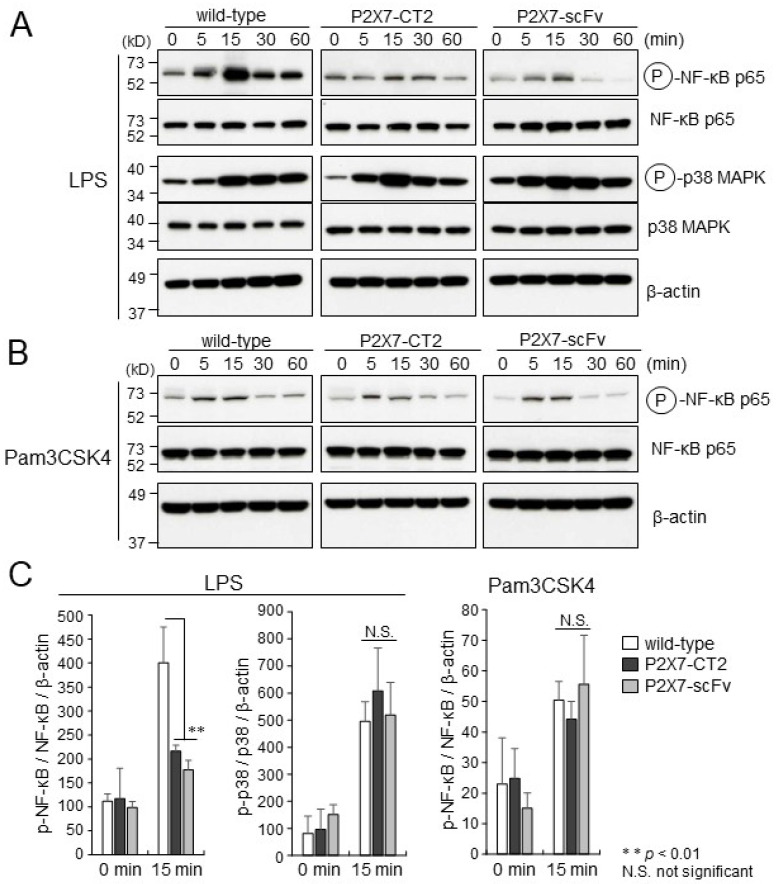
Phosphorylation of NF-κB and p38 MAPK induced by LPS or Pam3CSK4 stimulation. Wild-type, anti-P2X7-CT2 Tg, and anti-P2X7-scFv Tg BMDMs were stimulated with (**A**) LPS or (**B**) Pam3CSK4 for the time indicated and lysed. Cellular lysates were separated by SDS-PAGE and immunoblotted with anti-phospho-specific antibodies to NF-κB and p38 MAPK. Similar expression levels of NF-κB and p38 in wild-type, P2X7-CT2 Tg, and anti-P2X7-scFv Tg BMDMs were demonstrated by Western blotting with anti-NF-κB and anti-p38 antibodies. Equal protein loading was confirmed using an anti-β-actin antibody. Full-length gels/blots are presented in Supplementary Figure S2. Immunoblots are representative of three independent experiments. (**C**) The band intensities were measured with ImageJ software version 1.54g. Levels of total NK-κB and p38 MAPK were normalized to the level of β-actin; levels of phosphorylated NK-κB and p38 MAPK were normalized to the level of total NK-κB and p38 MAPK, respectively. ** *p* < 0.01. Error bars represent SE (*n*  =  3).

P2X7 overexpression reportedly enhances LPS-induced NF-κB activity, suggesting that P2X7 might be involved in the LPS-induced NF-κB activation pathway [22]. To assess the effects of P2X7-CT2 or anti-P2X7-scFv expression on NF-κB activation induced by LPS stimulation in macrophages, the extent of NF-κB phosphorylation upon LPS stimulation in wild-type, P2X7-CT2 Tg, and anti-P2X7-scFv Tg BMDMs were compared through Western blotting. In wild-type BMDMs, NF-κB p65 (Ser-536) was strongly phosphorylated after 15 min of LPS stimulation. In contrast, NF-κB p65 phosphorylation in P2X7-CT2 Tg and anti-P2X7-scFv Tg BMDMs was maintained at lower levels (Figure 2A). Expression intensity analysis showed the levels of NF-κB p65 phosphorylation in P2X7-CT2 Tg and anti-P2X7-scFv Tg BMDMs were approximately half that of wild-type BMDMs after 15 min of LPS stimulation (Figure 2C). The expression levels of total NF-κB p65 protein were observed similar among wild-type, P2X7-CT2 Tg, and anti-P2X7-scFv Tg BMDMs (Figure 2A). These findings suggest that the expression of the P2X7 C-terminal domain (P2X7-CT2) and anti-P2X7-scFv impairs NF-κB activation mediated by LPS stimulation. The phosphorylation profiles of p38 mitogen-activated protein kinase (MAPK) upon LPS stimulation were comparable among wild-type, P2X7-CT2 Tg, and anti-P2X7-scFv Tg BMDMs (Figure 2A,C), suggesting that P2X7-CT2 or anti-P2X7-scFv expression has an effect on the activation of NF-κB but not that of p38 MAPK, following LPS stimulation in BMDMs.

To examine the effects of P2X7-CT2 or anti-P2X7-scFv expression on the TLR2 signaling pathway in macrophages, wild-type, P2X7-CT2 Tg, and anti-P2X7-scFv Tg BMDMs were stimulated with Pam3CSK4 in vitro, after which the extent of NF-κB phosphorylation was determined by Western blotting. The equivalent phosphorylation profiles for NF-κB upon Pam3CSK4 stimulation were observed in wild-type, P2X7-CT2 Tg, and anti-P2X7-scFv Tg BMDMs (Figure 2B,C). These findings indicate that P2X7-CT2 or anti-P2X7-scFv expression specifically inhibits TLR4 signaling-induced NF-κB activation but does not affect the TLR2 signaling cascade in macrophages.

### 2.3. Impaired Cytokine Production in P2X7-CT2 Tg and Anti-P2X7-scFv Tg BMDMs upon LPS Stimulation

**Figure 3:**
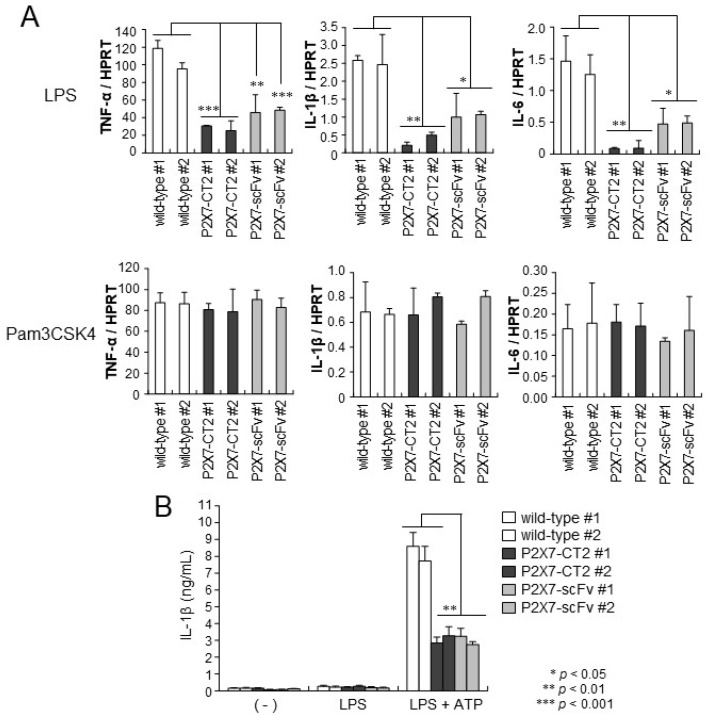
LPS-stimulated cytokine expression and production in BMDMs. (**A**) Quantitative real-time PCR was performed on RNA derived from wild-type, P2X7-CT2 Tg, and anti-P2X7scFv Tg BMDMs activated by LPS or Pam3CSK4 stimulation. Expression levels are shown relative to control HPRT. Values represent means ± SE of triplicate assays. (**B**) Wild-type, P2X7-CT2 Tg, and anti-P2X7-scFv Tg BMDMs were pretreated with or without LPS for 4 h, followed by treatment with or without ATP for 1 h. The culture supernatants were analyzed by ELISA to measure IL-1β levels. Values represent means ± SE of triplicate cultures. The profiles are typical examples of at least three independent experiments. #1 and #2 were clones each independently isolated from wild-type, P2X7-CT2 Tg, and anti-P2X7-scFv Tg mice. One-way ANOVA was used to calculate *p* values when compared to wild-type. * *p* < 0.05, ** *p* < 0.01, *** *p* < 0.001.

In activated macrophages, phosphorylation of NF-κB is essential for producing various inflammatory cytokines [23]. To evaluate the effects of P2X7-CT2 or anti-P2X7-scFv expression on the LPS signaling pathway, RNA was isolated from LPS-activated wild-type, P2X7-CT2 Tg, and anti-P2X7-scFv Tg BMDMs and analyzed by quantitative real-time PCR. TNF-α, IL-1β, and IL-6 mRNA levels were markedly upregulated upon LPS stimulation in wild-type BMDMs. In contrast, P2X7-CT2 Tg and anti-P2X7-scFv BMDMs showed approximately a quarter of the levels of TNF-α and IL-1β and a sixth of IL-6 transcription levels (Figure 3A). On the other hand, the levels of TNF-α, IL-1β, and IL-6 transcription upon Pam3CSK4 stimulation were similar among wild-type, P2X7-CT2 Tg, and anti-P2X7-scFv Tg BMDMs (Figure 3A). These results confirmed that the P2X7 C-terminal domain is crucial for the production of inflammatory cytokines following TLR4- but not TLR2-stimulation in macrophages.

Furthermore, we investigated the effects of P2X7-CT2 or anti-P2X7-scFv expression on ATP-induced IL-1β release from BMDMs using ELISA. In contrast to the high levels of IL-1β secretion induced by LPS-primed ATP stimulation in wild-type BMDMs, P2X7-CT2 Tg and anti-P2X7-scFv Tg BMDMs showed impaired ATP-induced IL-1β secretion (Figure 3B). The impairment of mature IL-1β secretion correlated with that of IL-1β transcription upon LPS stimulation in P2X7 CT2-Tg and anti-P2X7-scFv Tg BMDMs, implying that the P2X7 C-terminal domain plays a critical role in TLR4 signaling in macrophages.

### 2.4. P2X7 C-Terminal Domain Associates with MyD88 in BMDMs and Interacts with TLR4 C-Terminal Region in in Vitro Binding Assay

**Figure 4:**
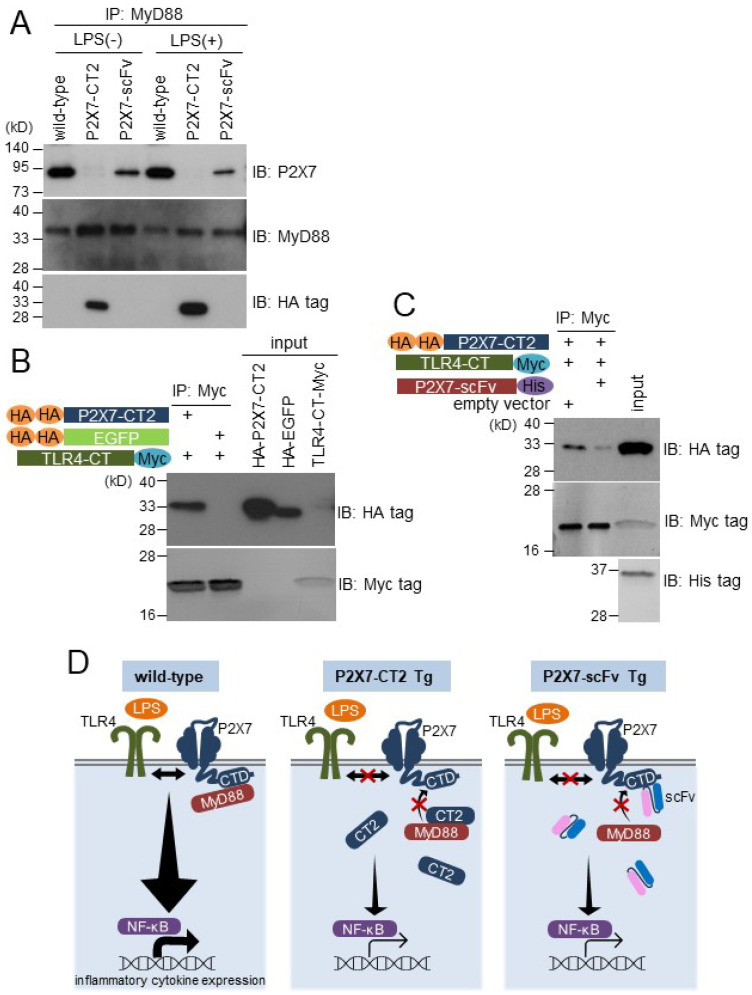
Interactions between the P2X7 C-terminal domain and MyD88 or TLR4 C-terminal region were inhibited by the P2X7 C-terminal domain or anti-P2X7-scFv expression. (**A**) Wild-type, P2X7-CT2 Tg, and anti-P2X7-scFv Tg BMDMs were cultured with or without LPS, and cell lysates were immunoprecipitated with anti-MyD88 antibodies. Immunocomplexes were immunoblotted with anti-P2X7, anti-MyD88, and anti-HA tag antibodies. (**B**) and (**C**) DNA constructs encoding HA-P2X7-CT2, HA-EGFP, TLR4-CT-Myc, and anti-P2X7-scFv-His were transiently transfected into DO-11.10 cells. The lysates were mixed and immunoprecipitated with an anti-Myc tag antibody. Immunocomplexes were analyzed by Western blotting with an anti-HA tag, anti-Myc tag, or anti-His tag antibody. Full-length gels/blots are presented in Supplementary Figure S3. All results are representative of three independent experiments. (**D**) Schematic showing the relationship between P2X7, MyD88, and TLR4 in wild-type BMDMs and the interference in their association caused by overexpression of P2X7-CT2 or anti-P2X7-scFv in the Tg BMDMs.

The interaction between P2X7 and MyD88 has been observed in transient co-expression experiments using HEK293T cells [20]. To investigate whether endogenous P2X7 could bind MyD88 in BMDMs, wild-type, P2X7-CT2 Tg, and anti-P2X7-scFv Tg BMDMs were lysed and immunoprecipitated with anti-MyD88 antibody. The immunocomplexes were immunoblotted using an anti-P2X7 antibody. A specific interaction between endogenous P2X7 and MyD88 was clearly observed in wild-type BMDMs regardless of the presence or absence of LPS (Figure 4A). On the other hand, the interaction between P2X7 and MyD88 was hindered in P2X7-CT2 Tg and anti-P2X7-scFv Tg BMDMs (Figure 4A). Expression levels of endogenous P2X7 and MyD88 were comparable among wild-type, P2X7-CT2 Tg, and anti-P2X7-scFv Tg BMDMs (Figure 1D and Figure 4A). These results suggest that P2X7-CT2 or anti-P2X7-scFv expression inhibits the specific interaction between endogenous P2X7 and MyD88 in BMDMs.

MyD88 is an adaptor molecule that forms signaling complexes downstream of TLR4, leading to NF-κB activation in response to LPS stimulation [24,25]. To investigate whether the P2X7 C-terminal domain interacts with TLR4, DNA constructs encoding HA-P2X7-CT2, HA-EGFP, and TLR4-CT-Myc were transiently transfected into DO-11.10 cells, and each of the cell lysates was mixed and immunoprecipitated with anti-Myc tag antibody. Strong binding was observed between HA-P2X7-CT2 and TLR4-CT-Myc, whereas control HA-EGFP did not bind TLR4-CT-Myc (Figure 4B). Furthermore, to investigate the inhibitory effect of anti-P2X7-scFv on the association between the P2X7 C-terminal domain and TLR4 C-terminal region, DNA constructs encoding HA-P2X7-CT2, TLR4-CT-Myc, and anti-P2X7-scFv-His were transiently transfected into DO-11.10 cells, and each of the cell lysates was mixed and immunoprecipitated with anti-Myc tag antibody. In the absence of anti-P2X7-scFv, P2X7-CT2 strongly interacted with TLR4-CT. However, in the presence of anti-P2X7-scFv, the binding between P2X7-CT2 and TLR4-CT was severely inhibited, probably by scFv masking the TLR4 binding site in the P2X7 C-terminal domain (Figure 4C). It remains unclear whether the P2X7 C-terminal domain and the TLR4 C-terminal region associate directly or via MyD88, because DO-11.10 cells contain endogenous MyD88. These observations suggest that P2X7 plays a pivotal role in the LPS-induced activation of NF-κB accompanied by the recruitment of MyD88 and TLR4 through its intracellular C-terminal domain in macrophages. Overexpression of P2X7-CT2 or anti-P2X7-scFv may interfere with the complex formation of P2X7-MyD88-TLR4, resulting in impaired LPS signaling in the Tg BMDMs (Figure 4D).

## 3. Discussion

In this study, we demonstrated that BMDM cell lines established from transgenic mice overexpressing the P2X7 C-terminal domain or anti-P2X7 C-terminal domain scFv intrabody displayed strongly impaired inflammatory cytokine production upon LPS stimulation. These results suggest that P2X7 mediates macrophage LPS signaling through its C-terminal domain.

Recently, P2X7 has been suggested to play a pivotal role in the inflammatory response of immune cells [26]. Using gene-transfected HEK293T cells, Liu et al. demonstrated that P2X7 bound MyD88 and enhanced MyD88-induced NF-κB activation [20]. In the present study, the P2X7 C-terminal domain was confirmed to associate with MyD88 with/without LPS stimulation in BMDMs (Figure 4A). This interaction was inhibited by the P2X7 C-terminal domain and anti-P2X7 C-terminal domain scFv intrabody overexpression in Tg BMDMs (Figure 4A), suggesting that the P2X7 C-terminal domain is important for the interaction between P2X7 and MyD88 in macrophages. Furthermore, the in vitro binding assay demonstrated that the P2X7 C-terminal domain interacts with the TLR4 intracellular C-terminal region (Figure 4B). This interaction was inhibited by the anti-P2X7 scFv, which masked the TLR4 binding site in the P2X7 C-terminal domain (Figure 4C). However, a specific interaction between P2X7 and endogenous TLR4 in BMDMs could not be observed. Further investigations are necessary to understand the mechanisms of the P2X7-MyD88-TLR4 signaling complex.

NF-κB p65 phosphorylation at Ser-536 is essential for the nuclear translocation of NF-κB and induction of inflammatory cytokine gene expression [27,28]. P2X7 C-terminal domain and anti-P2X7 C-terminus scFv overexpression strongly inhibited NF-κB p65 phosphorylation upon LPS stimulation in Tg BMDMs (Figure 2A,C). Similarly, LPS-induced TNF-α, IL-1β, and IL-6 gene transcription was significantly reduced in Tg BMDMs (Figure 3A). However, NF-κB activation and inflammatory cytokine gene transcription induced by Pam3CSK4, a TLR2 ligand, were not affected (Figure 2B,C and Figure 3A). Like TLR4 signaling, TLR2-dependent signaling cascades ultimately lead to MyD88- and MAL/TIRAP-dependent NF-κB activation [29]. These findings suggest that the P2X7- MyD88 complex may specifically associate with the TLR4 intracellular C-terminal region and modulate inflammatory responses upon LPS stimulation in macrophages.

P2X7 is an ATP-gated cation channel. The overexpression of the P2X7 C-terminal domain or anti-P2X7 C-terminus scFv intrabody did not affect ATP-induced Ca^2+^ influx in Tg BMDMs (Supplementary Figure S4). These results indicate that endogenous P2X7 correctly localizes on the cell surface membrane and normally functions as an ATP-gated cation channel without being affected by P2X7 C-terminal domain and anti-P2X7 C-terminal domain scFv intrabody overexpression in Tg BMDMs. Taken together, the P2X7 C-terminal domain plays a critical role in LPS-induced signal transduction but not in the ATP-gated cation channel in macrophages.

In conclusion, our findings suggest that P2X7 C-terminal domain function knockdown by P2X7 C-terminal domain and anti-P2X7 C-terminal domain scFv intrabody overexpression can inhibit inflammatory cytokine production upon LPS stimulation in Tg BMDMs. Moreover, P2X7 plays an important role in the inflammatory response to LPS by complex formation with MyD88 and TLR4 through its intracellular C-terminal domain in macrophages. Thus, designing drugs with the biological features of anti-P2X7 C-terminal domain scFv intrabody may provide novel anti-inflammatory agents. Recent research has identified and characterized several signaling molecules involved in TLR signaling, contributing to an increased understanding of the activation mechanisms of innate immunity in pathogen recognition. However, due to the multiplexity of the TLR signaling cascades, signal transduction mechanisms via TLRs have not been fully elucidated. Further investigation and identification of new mediators in TLR signal transduction will help to clarify the mechanisms of innate immune responses to microbial pathogens.

## 4. Materials and Methods

### 4.1. Plasmid Construction

A cDNA fragment of the mouse P2X7-C-terminal domain (amino acids 392–595, designated P2X7-CT2) was generated by PCR using the sense primer 5′-CGAATGCGGCCGCAATGGAACCCAAGCCGACG-3′ and antisense primer 5′-CGAATGCGGCCGCTCAGTAGGGATACTTGAAGC-3′. The EGFP fragment was amplified from a pEGFP vector (Clontech, Mountain View, CA, USA) using PCR and the sense primer 5′-CGAATGCGGCCGCAGTACCGGTCGCCACCATGGTG-3′ and antisense primer 5′-CGAATGCGGCCGCTTTACTTGTACAGCTCGTC-3′. The double (D)-HA tag (YPYDVPDYAYPYDVPDYA) was inserted into the *Hind*III-*Not*I site of the pCAGGS-MCS expression vector. Next, PCR-amplified P2X7-CT2 and EGFP fragments were digested using NotI and cloned into the pCAGGS/(D)-HA vector, resulting in the fusion of P2X7-CT2 and EGFP with the (D)-HA tag at the N-terminus. Furthermore, the cDNA fragment of P2X7-CT2, digested using *Not*I, was cloned into the *Not*I site of the pGEX-4T-2 vector (GE Healthcare, Buckinghamshire, UK). The GST-P2X7-CT2 fusion protein was produced in BL21 *Escherichia coli* cells and purified on a Glutathione-Sepharose 4B Affinity Chromatography Column (GE Healthcare) according to the manufacturer’s instructions. Anti-P2X7 mAbs (clones 5 and 18) were prepared from mice immunized using GST-P2X7-CT2 fusion protein by the conventional procedure [30].

A cDNA fragment of the mouse TLR4 C-terminal intracellular domain (amino acids 660–835, designated TLR4-CT) was generated by PCR using the sense primer 5′-CGAATGCGGCCGCGCCACCATGAGTCAGAATGAGGACTGGGTGAG-3′ and antisense primer 5′-CGAATGCGGCCGCCGGTCCAAGTTGCCGTTTCTTGTTC-3′, digested using *Not*I, and cloned into the pCAGGS-MCS expression vector [31,32]. The Myc tag (EQKLISEEDL) was inserted into the *Xba*I/*EcoR*I site of the pCAG/TLR4-CT construct, resulting in the fusion of TLR4-CT with the Myc tag at the C-terminus.

A GST fragment was amplified from the pGEX-4T-2 vector (GE Healthcare) using PCR and the sense primer 5′-GGATTCCATATGTCCCCTATACTAGGTTATTGG-3′ and antisense primer 5′-GTCAGTCAGTCACGATGCGGCCGCTCGAGTCG-3′. A P2X7-CT2 fragment was generated by PCR using the sense primer 5′-GGATTCCATATGGAACCCAAGCCGACG-3′ and antisense primer 5′-CGAATGCGGCCGCGTAGGGATACTTGAAGC-3′. cDNA fragments of GST and P2X7-CT2 were digested using *Nde*I and *Not*I and cloned into the *Nde*I/*Not*I site of the pET-23(a) vector (Novagen, Madison, WI, USA). The P2X7-CT2-His and GST-His fusion proteins were produced in BL21 *Escherichia coli* cells and purified on a Ni-Sepharose 6 Fast Flow Affinity Chromatography Column (GE Healthcare) according to the manufacturer’s instructions.

### 4.2. Cloning and Construction of Anti-P2X7-scFv

Total RNA from hybridoma-producing anti-P2X7 mAb (clone 5) was reverse-transcribed using a SMARTTM RACE cDNA Amplification Kit (Clontech). The cDNA fragments of the V_H_ and V_L_ regions were amplified by PCR using appropriate primers and then mixed and assembled into the scFv by four-step PCR amplification using appropriate primers containing linker sequences (Supplementary Figure S5 and Supplementary Table S1). The resulting fragment was digested using *Not*I/*Xba*I and cloned into the pCAGGS-MCS expression vector. A Myc (EQKLISEEDL) or His tag (6× His) was inserted into the *Xba*I/*EcoR*I site of the pCAG/anti-P2X7-scFv construct, resulting in the fusion of anti-P2X7-scFv with the Myc or His tag at the C-terminus. The GenBank/EMBL/DDBJ accession numbers for the sequences of the cDNAs encoding V_H_ and V_L_ are LC770195 and LC770196, respectively.

### 4.3. Generation of Transgenic Mice

The transgenes were cut out from the expression vectors pCAG/(D)HA-P2X7-CT2 and pCAG/anti-P2X7-scFv-Myc using *Sal*I/*Nhe*I restriction enzymes and purified using agarose gel electrophoresis and a QIAquick Gel Extraction kit (Qiagen, Hilden, Germany). The transgenes were adjusted to a final concentration of 3 µg/mL and microinjected into the fertilized egg pronuclei of inbred C57BL/6 mice. Next, the injected eggs were transferred into the oviducts of pseudopregnant female ICR mice. Procedures involving animal subjects were approved by the Institutional Animal Care and Use Committee at the National Institute of Agrobiological Sciences (approval ID: H28-006).

### 4.4. Cells and Electroporation

The murine T-cell hybridoma DO-11.10 and hybridoma cells producing anti-P2X7 antibodies were maintained in RPMI1640 medium supplemented with 100 U/mL penicillin, 100 µg/mL streptomycin, 4 mM L-glutamine, 10 mM HEPES (all obtained from Nacalai Tesque, Kyoto, Japan), and 10% fetal bovine serum. After being adjusted to 5 × 10^6^ cells/400 µL in a culture medium containing 1.25% dimethyl sulfoxide per cuvette, DO-11.10 cells were electroporated using a Gene Pulser (Bio-Rad Laboratories, Hercules, CA, USA) with 20 µg plasmid DNA at 290 V and 960 µF.

### 4.5. Establishment of BMDM Cell Lines

BMDMs were isolated from the bone marrow of P2X7-CT2 Tg mice and anti-P2X7-scFv Tg mice and maintained as described previously [33]. The procedure to immortalize BMDMs has also been described previously [33].

### 4.6. Immunocytochemistry

BMDMs were seeded in eight-well chamber slides (5 × 10^4^ cells/well) and fixed with 10% formalin in PBS for 30 min at 4 °C. After fixing, the cells were washed with cold PBS and then incubated with 1% Triton X in PBS for 30 min at 4 °C. Thereafter, the cells were washed with cold PBS and incubated with Dako REAL Peroxidase-Blocking Solution (Dako, Glostrup, Denmark) for 10 min at 25 °C to block endogenous peroxidase activity, followed by applying blocking solution with 2% BSA in PBS for 30 min at 25 °C. Next, cells were incubated with primary antibodies against F4/80 (rat, clone CI:A3-1) (BioLegend, San Diego, CA, USA) or control rat IgG (sc-2026, Santa Cruz Biotechnology, Dallas, TX, USA) for 1 h at 25 °C. Dako REAL EnVision-HRP (Dako) was applied as a secondary antibody for 1 h at 25 °C. Finally, a colorimetric substrate, 3,3′-diaminobenzidine tetrahydrochloride (Dako), was applied according to the manufacturer’s instructions. After additional washing with distilled water, the slides were dehydrated and mounted on coverslips using Mount-Quick (Daido Sangyo Co. Ltd., Toda, Japan).

### 4.7. FACS Analysis

BMDMs (1 × 10^6^ cells) were incubated with 10 μg/mL Fc Block (rat anti-CD16/32 monoclonal antibody, clone 2.4G2; BD Pharmingen, San Diego, CA, USA) for 10 min at 4 °C and then stained with phycoerythrin-conjugated anti-CD11b (rat, clone M1/70, BioLegend), anti-F4/80 (rat, clone CI:A3-1, BioLegend), anti-TLR4 antibody (rat, clone MTS510, BioLegend), or the isotype control antibodies rat IgG2a (clone RTK2758) and rat IgG2b (clone G013B8, BioLegend) for 60 min at 4 °C. After washing with PBS, the cells were analyzed by flow cytometry (EPICS XL; Beckman Coulter, Brea, CA, USA).

### 4.8. Western Blot Analysis

BMDMs were lysed using an SDS sample buffer and boiled for 10 min. Thereafter, the cell lysates were separated by 12% SDS-PAGE and transferred to a polyvinylidene difluoride membrane (Bio-Rad Laboratories). Next, the membrane was incubated with Blocking One (Nacalai Tesque) and probed with anti-P2X7 pAb (Alomone (#APR-004), Jerusalem, Israel) or biotin-conjugated anti-Myc tag mAb (MBL, Minato-ku, Japan), followed by HRP-conjugated anti-rabbit IgG (Dako) or HRP-conjugated avidin (Biolegend). Immunoreactive proteins were detected using Chemi-Lumi One L or Chemi-Lumi One Super (Nacalai Tesque).

BMDMs were activated using LPS (1 μg/mL; ultra-pure E. coli 0111: B4 LPS) or Pam3CSK4 (1 μg/mL) (all obtained from InvivoGen, San Diego, CA, USA) for different time intervals at 37 °C. After washing with PBS, the activated cells were lysed with SDS sample buffer and boiled for 10 min. Subsequently, the cell lysates were immunoblotted with anti-phospho-NF-κB p65 (Ser-536) antibody, anti-NF-κB p65 antibody, anti-phospho-p38 antibody, anti-p38 antibody, or anti-β-actin antibody (Cell Signaling Technology, Danvers, MA, USA). The band intensities of protein expression were quantified by densitometric analysis using ImageJ software version 1.54g (National Institutes of Health). Bands were selected by a rectangular selection and plotted. Areas were measured by the wand tool and normalized for the final statistics.

### 4.9. Quantitative Real-Time PCR

BMDMs were cultured with LPS (1 μg/mL) or Pam3CSK4 (1 μg/mL) at 37 °C for 5 h and then lysed using a Real-Time Ready Cell Lysis Kit (Roche Diagnostics, Basel, Switzerland). cDNA was obtained using Transcriptor Universal cDNA Master (Roche Diagnostics) according to the manufacturer’s instructions. Real-time PCR for mouse inflammatory cytokines was performed in a LightCycler 1.5 PCR System (Roche). cDNA was amplified using a LightCycler TaqMan Master Kit (Roche) with Universal ProbeLibrary Probe #78 (Roche) and specific primer sets for TNF-α, IL-1β, IL-6, and HPRT. HPRT was utilized as a standard using Universal ProbeLibrary Probe #22 (Roche) and a specific primer set. The primer sequences are listed in Supplementary Table S2.

### 4.10. ELISA

BMDMs were seeded in 48-well plates (1 × 10^5^ cells/500 μL/well) and cultured with or without LPS (1 μg/mL) at 37 °C for 4 h and then further cultured with or without ATP (2 mM) at 37 °C for 1 h. The levels of IL-1β in the culture supernatant were quantified in triplicate using ELISA MAXTM Set Deluxe (BioLegend) according to the manufacturer’s instructions.

### 4.11. Immunoprecipitation Assay

BMDMs were activated with or without LPS (1 μg/mL) for 15 min and then lysed with RIPA buffer (Nacalai Tesque) on ice for 1 h. Following centrifugation at 10,000× g for 10 min at 4 °C, the lysates were incubated with Precleaning Matrix C (Santa Cruz Biotechnology) for 1 h at 4 °C to remove nonspecifically binding proteins. Afterward, the cleared lysates were incubated with anti-MyD88 goat pAb (Santa Cruz Biotechnology) or anti-P2X7 mAb (clone 18) and pulled down using Exacta Cruz C IP-Matrix Beads (Santa Cruz Biotechnology). Thereafter, immunocomplexes were washed five times with PBS and resuspended in an SDS sample buffer and boiled for 10 min. Next, the immunocomplexes were immunoblotted with rabbit anti-P2X7 pAb (5 μg/mL, raised against a synthetic peptide representing P2X7 residue 72–87), anti-MyD88 pAb (Cell Signaling Technology), anti-HA tag pAb, or biotin-conjugated anti-Myc tag mAb (MBL).

DNA constructs encoding HA-P2X7-CT2, HA-EGFP, TLR4-CT-Myc, and anti-P2X7-scFv-His were transiently transfected into DO-11.10 cells. After washing with PBS, the gene-transfected DO-11.10 cells were lysed with RIPA buffer on ice for 1 h. Subsequently, the lysates were centrifuged at 10,000× g for 10 min at 4 °C, after which each of the supernatants was mixed and incubated at 4 °C overnight. Next, protein complexes were obtained using a Myc Tagged Protein Purification Kit (MBL) according to the manufacturer’s instruction, lysed with SDS sample buffer, and immunoblotted with anti-HA tag pAb, anti-Myc tag pAb, or anti-His tag pAb (MBL).

### 4.12. Statistical Analysis

Real-time PCR and ELISA were performed in triplicate for each experimental condition. Data are expressed as mean ± SE of the mean. *p*-values were calculated using one-way ANOVA with GraphPad Prism 6 (GraphPad Software, La Jolla, CA, USA). Statistical significance was set at * *p* < 0.05, ** *p* < 0.01, and *** *p* < 0.001.

## Data Availability

The sequences of the cDNAs encoding anti-P2X7 mAb (clone 5) have been deposited in GenBank/EMBL/DDBJ under the accession numbers LC770195 for V_H_ and LC770196 for V_L_.

## Supplementary Materials

The following supporting information can be downloaded at: https://www.mdpi.com/article/10.3390/ijms26031178/s1. Reference [34] has been cited in Supplementary Materials file.
