# Supplementary material for: Impaired LPS Signaling in Macrophages Overexpressing the P2X7 C-Terminal Domain or Anti-P2X7 C-Terminal Domain Intrabody
Source: Int J Mol Sci. 2025 Jan 29;26(3):1178. doi: 10.3390/ijms26031178 (PMC11818346; doi:10.3390/ijms26031178)
Supplement: Supplementary file 1 [file ijms-26-01178-s001.zip › ijms-3404280-supplementary.pdf]

International Journal of *Molecular Sciences*

MDPI

Article

Impaired LPS Signaling in Macrophages Overexpressing the P2X7 C-Terminal Domain or Anti-P2X7 C-Terminal Domain Intrabody

Chisato Sakuma 1, Takato Takenouchi 2 and Mitsuru Sato 1, *

1 Silkworm Research Group, Division of Silk-Producing Insect Biotechnology, Institute of Agrobiological Sciences, National Agriculture and Food Research Organization, Tsukuba, Ibaraki 305-8634, Japan; sakumac590@affrc.go.jp
2 Animal Model Development Group, Division of Biomaterial Sciences, Institute of Agrobiological Sciences, National Agriculture and Food Research Organization, Tsukuba, Ibaraki 305-8634, Japan; ttakenou@af-frc.go.jp
* Correspondence: Correspondence: mitsuru.sato@affrc.go.jp; Tel.: +81-29-838-6041

Supplementary Material

1. Supplementary Figures and Tables

1.1. Supplementary Figures

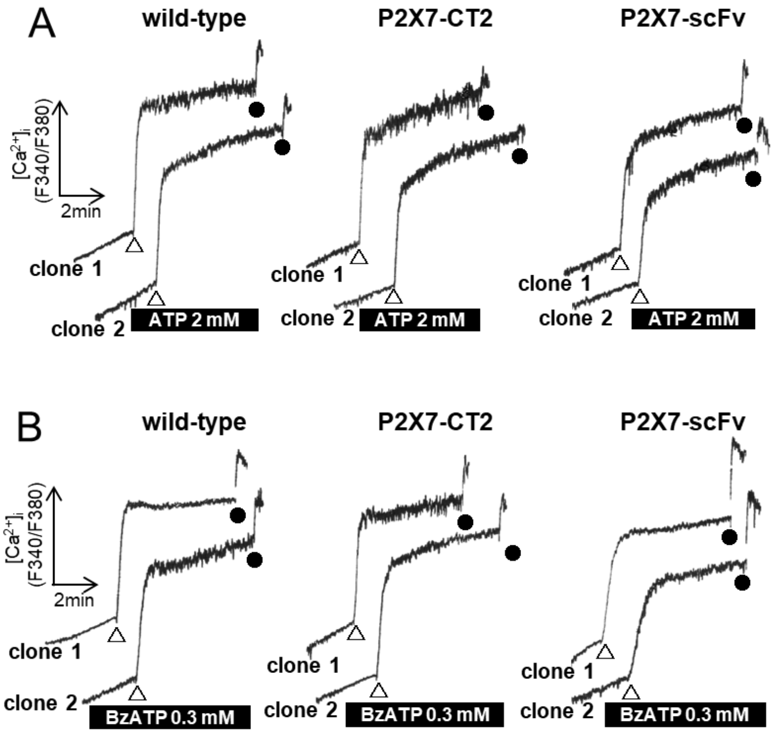

Supplementary Figure S1. P2X7 mediated the sustained Ca^{2+} influx observed in bone marrow-derived macrophages (BMDMs). Wild-type, P2X7-CT2 Tg, and anti-P2X7-scFv Tg BMDMs were cultured in the presence of (A) ATP or (B) BzATP (Δ) and $[Ca^{2+}]i$ was measured by monitoring fura-2 fluorescence. At the end of each experiment, BMDMs were permeabilized by treatment with 0.2% Triton X-100, and the maximum fura-2 fluorescence was measured (closed circles). Clones #1 and #2 were each independently isolated from wild-type, P2X7-CT2 Tg, and anti-P2X7-scFv Tg mice. All results are representative of three independent experiments.

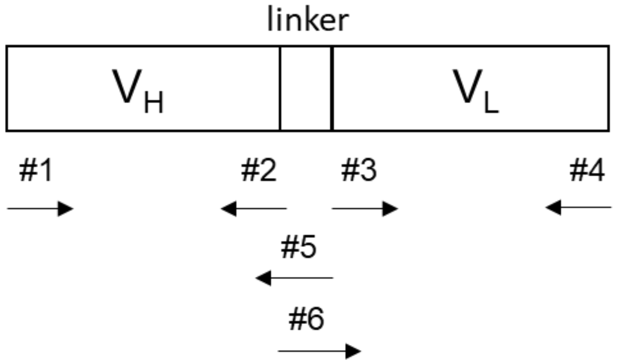

Supplementary Figure S2. Generation of anti-P2X7-scFv by cloning the variable region of immunoglobulin heavy and light chains from hybridoma cells producing anti-P2X7 monoclonal antibody. The arrows represent the primers used to amplify the sequences encoding antibody fragments.The anti-P2X7-mAb clone 5 isotype was identified as IgG2a and κ using an IsoStrip mouse monoclonal antibody isotyping kit (Roche Diagnostics, Mannheim, Germany). Four-step PCR was conducted to generate appropriate cDNA fragments encoding the V_H and V_L regions. Total RNA from hybridoma cells was reverse-transcribed using a SMARTTM RACE cDNA Amplification Kit (Clontech, Palo Alto, CA, USA). The cDNA fragments for the V_H and V_L regions were generated by PCR using isotype-specific primers (heavy chain, IgG2a: sense primer 5′-AAGCAGTGGTATCAACGCAGAG-TACGCG-3′ and antisense primer 5′-GGGCCCTCTGGGCTCAATTTTCTTGTC-3′; light chain, κ: sense primer 5′- AAGCAGTGGTATCAACGCAGAGTACGCG-3′ and antisense primer 5′-CCTGTT-GAAGCTCTTGACAATGGGTG-3′). The second PCR amplification was performed with the following primer sets: V_H, sense primer #1 and antisense primer #2; V_L, sense primer #3 and antisense primer #4. The third set of PCR products were amplified using the following primer sets: V_H-linker, sense primer #1 and antisense primer #5; linker-V_L, sense primer #6 and antisense primer #4. The third set of PCR products, V_H-linker and linker-V_L, were mixed, and then the fourth PCR amplification was performed using the following primer sets: sense primer #1 and antisense primer #4. The fourth set of PCR products were digested with *Not*I-*Xba*I and cloned into the pCAGGS-MCS expression vector.

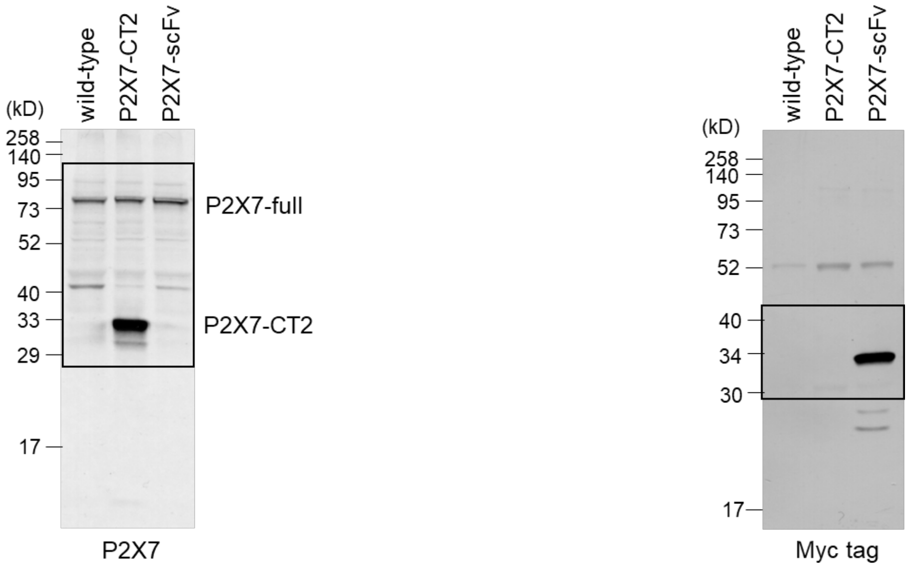

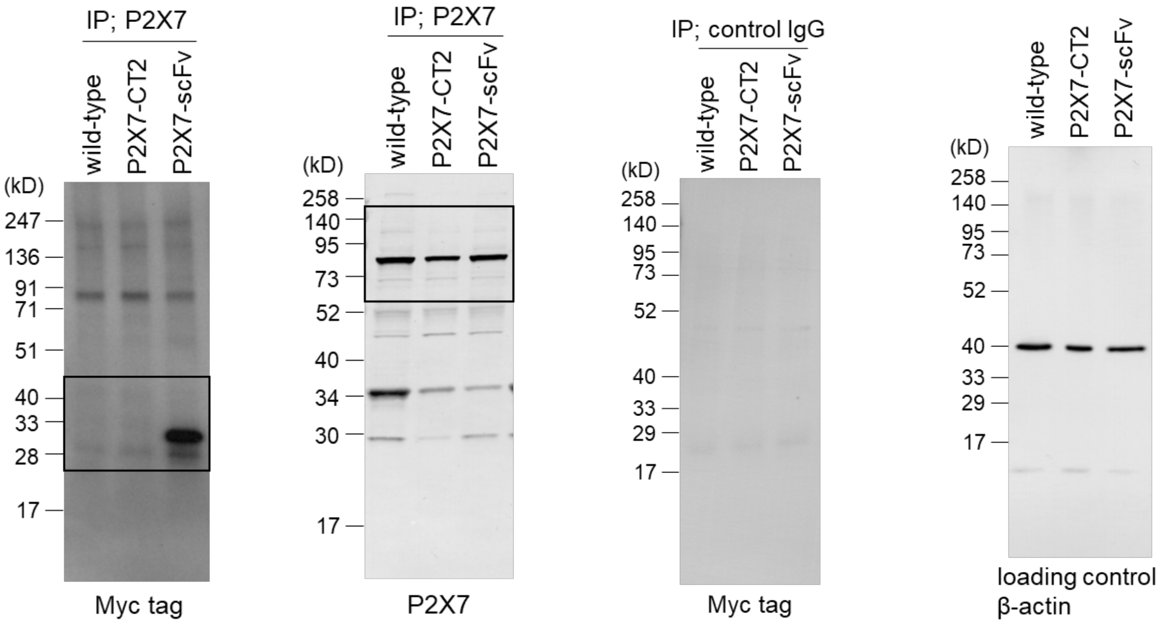

Supplementary Figure S3. Full-length gels/blots and control data for Figure 1. Western blots of wild-type, P2X7-CT2 Tg, and anti-P2X7-scFv Tg bone marrow-derived macrophages and immunocomplexes obtained from co-immunoprecipitation using anti-P2X7 antibody or control mouse IgG. The immunoblots were probed with an anti-P2X7, anti-Myc tag or anti-β-actin antibody. Insets indicate cropped blots in Figure 1C and D.

LPS

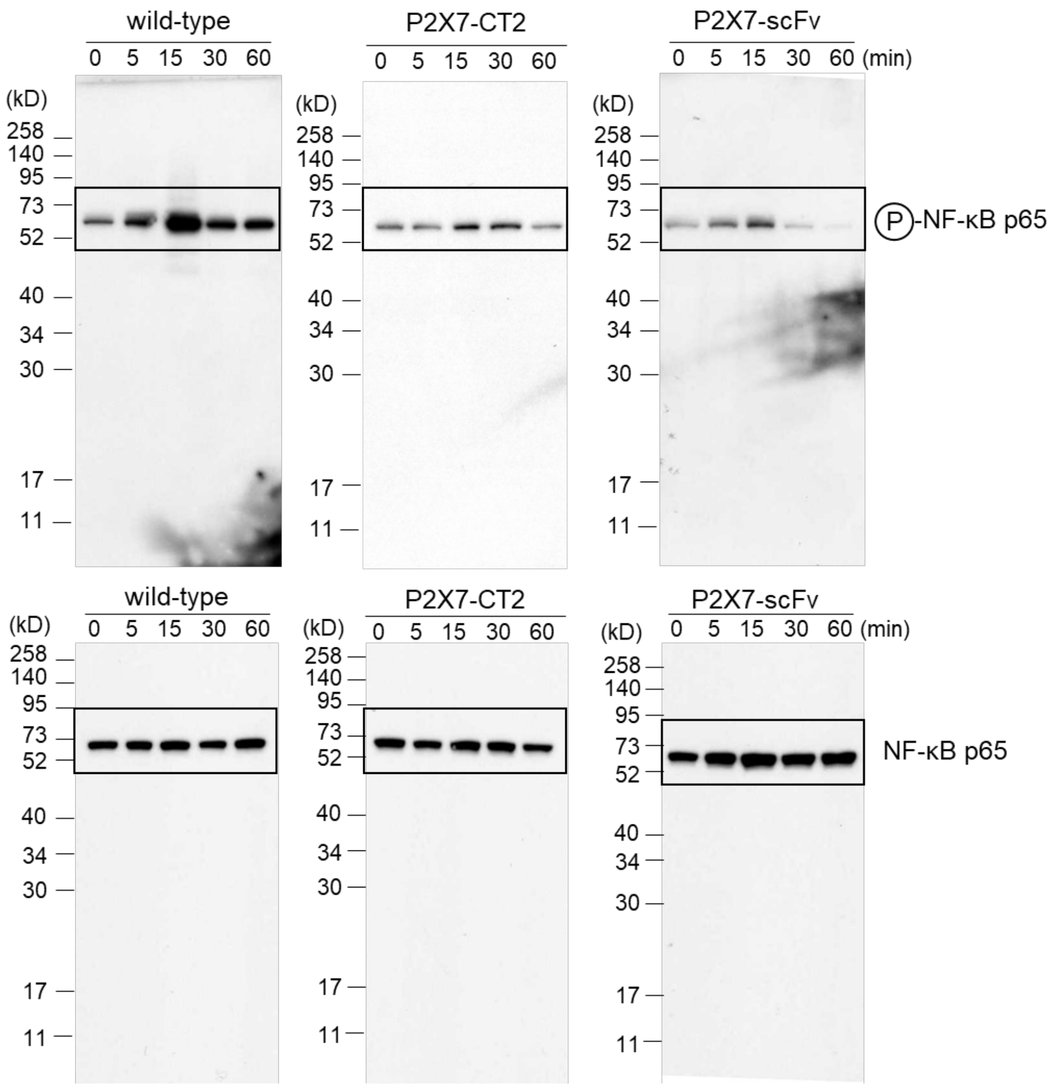
wild-type
P2X7-CT2
P2X7-scFv
0 5 15 30 60 (min)
(kD)
258
140
95
73
52
40
34
30
17
11
P-NF-κB p65
NF-κB p65

LPS

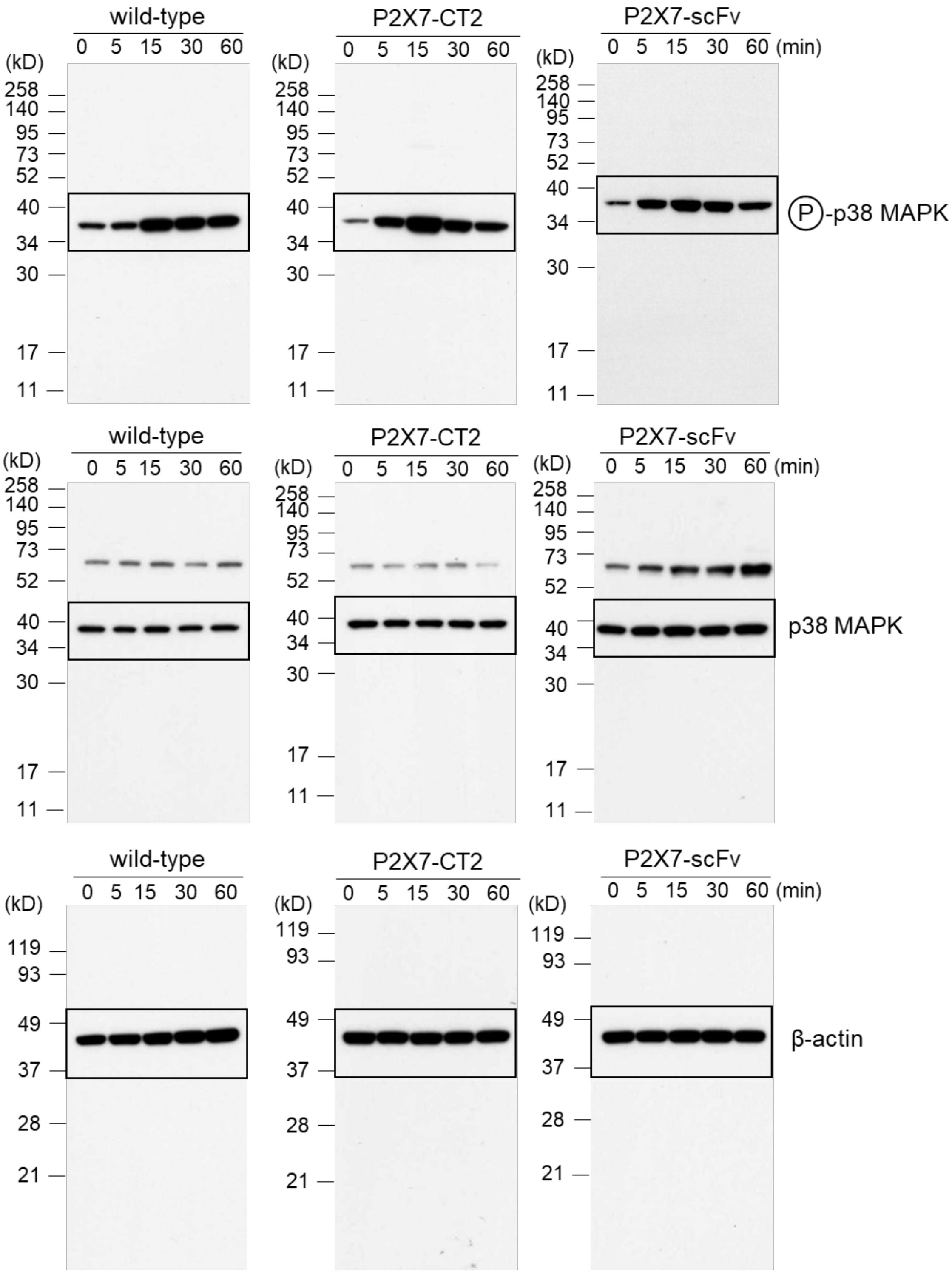
wild-type
P2X7-CT2
P2X7-scFv
0 5 15 30 60 (min)
(kD)
258
140
95
73
52
40
34
30
17
11
P-p38 MAPK
p38 MAPK
119
93
49
37
28
21
β-actin

Pam3CSK4

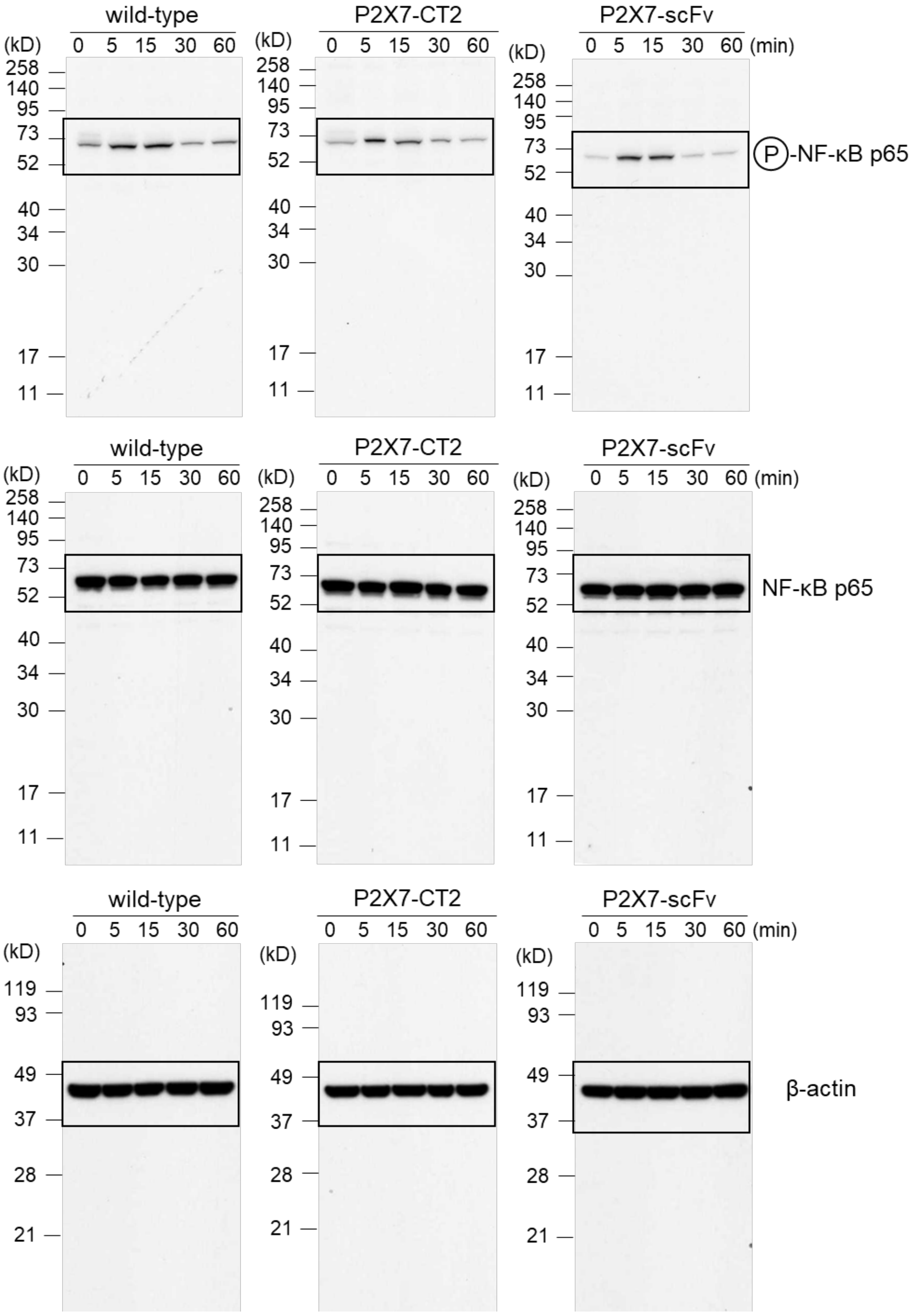

Supplementary Figure S4. Full-length gels/blots for Figure 2. Western blots of wild-type, P2X7-CT2 Tg, and anti-P2X7-scFv Tg bone marrow-derived macrophages stimulated with LPS or Pam3CSK4. The immunoblots were probed with anti-phospho-specific antibodies to NF-κB and p38 MAPK, anti-NF-κB, anti-p38 MAPK, or anti-β-actin antibodies. Insets indicate cropped blots in Figure 2A and B.

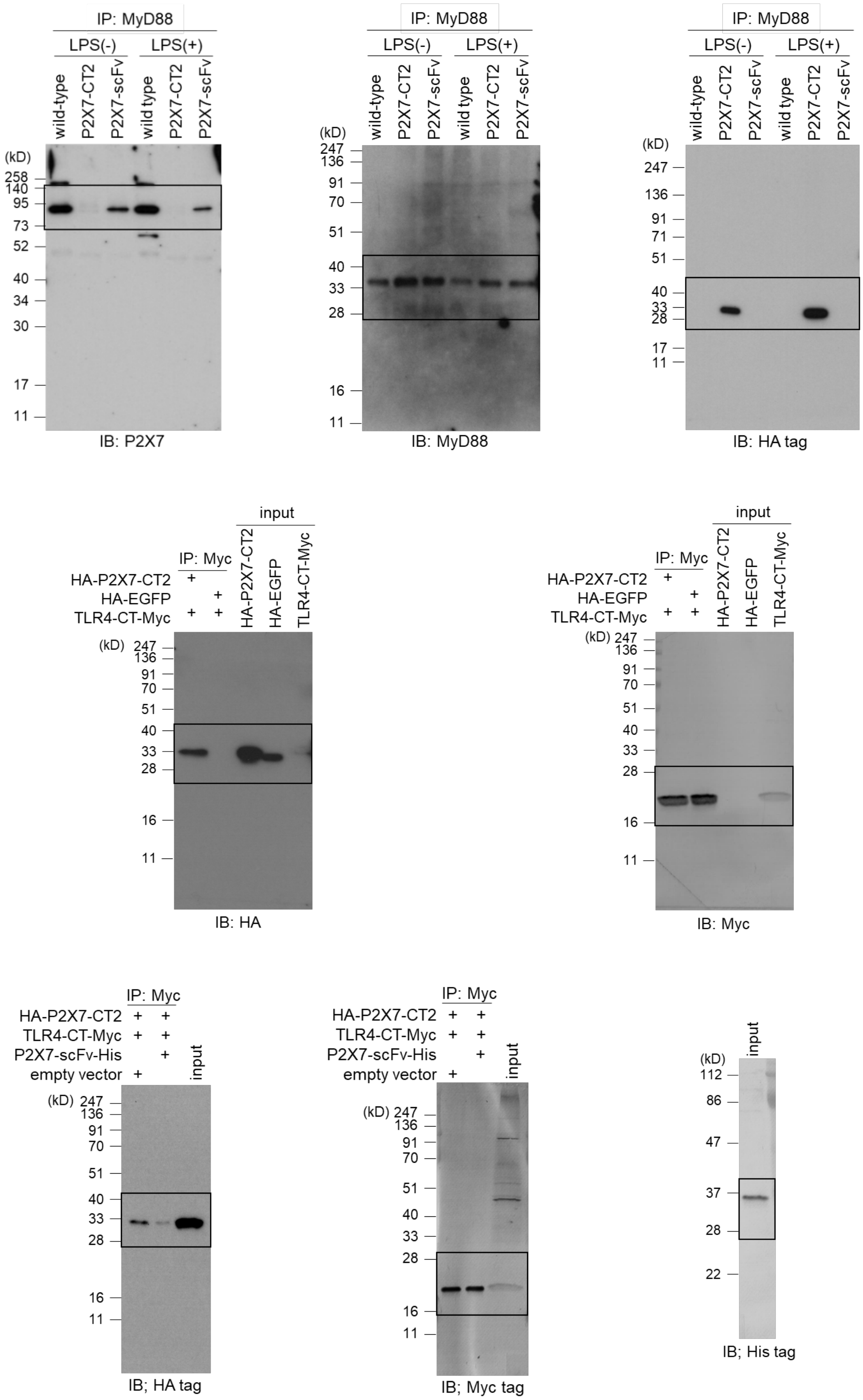
IP: MyD88
LPS(-)
LPS(+)
wild-type
P2X7-CT2
P2X7-scFv
wild type
P2X7-CT2
P2X7-scFv
(kD)
258
140
95
73
52
40
34
30
17
11
IB: P2X7
247
136
91
70
51
40
33
28
16
11
IB: MyD88
71
17
IB: HA tag
input
IP: Myc
HA-P2X7-CT2
HA-EGFP
TLR4-CT-Myc
IB: HA
IB: Myc
P2X7-scFv-His
empty vector
IB; HA tag
IB; Myc tag
112
86
47
37
22
IB; His tag

Supplementary Figure S5. Full-length gels/blots for Figure 4. Western blots of immunocomplexes obtained from co-immunoprecipitation using anti-MyD88 or anti-Myc tag antibodies. The immunoblots were probed with anti-P2X7, anti-MyD88, anti-HA tag, anti-Myc, or anti-His tag antibodies. Insets indicate cropped blots in Figure 4.

Materials and methods

Measurement of $[Ca^{2+}]i$

The $[Ca^{2+}]i$ of bone marrow-derived macrophages (BMDMs) induced by stimulation with a P2X7 agonist (ATP or BzATP) was measured by monitoring fura-2 fluorescence as described previously [1]. Fluorescence was measured at 500 nm [excitation wavelengths of 340 (F340) and 380 nm (F380)]. The ratio of the fluorescence intensities observed at the two aforementioned wavelengths (F340/F380) was used to indicate $[Ca^{2+}]i$.

Result

ATP-induced P2X7 mediates sustained Ca^{2+} influx in P2X7-CT2 Tg and anti-P2X7-scFv Tg BMDMs

Recent studies demonstrated that millimolar extracellular ATP concentrations induce sustained Ca^{2+} influx via P2X7 activation, while micromolar concentrations induce a transient increase in intracellular Ca^{2+} concentrations ($[Ca^{2+}]i$) by activating other types of P2 receptors [2]. To investigate whether P2X7-CT2 or anti-P2X7-scFv expression affects the cation channel function of endogenous P2X7, $[Ca^{2+}]i$ were measured in wild-type, P2X7-CT2 Tg, and anti-P2X7-scFv Tg BMDMs. Extracellular ATP at a concentration of 2 mM induced a sustained increase in $[Ca^{2+}]i$ in wild-type, P2X7-CT2 Tg, and anti-P2X7-scFv Tg BMDMs (clone #1, #2) (Supplementary Figure S5A). Similarly, BzATP, a potent P2X7 agonist, at a concentration of 0.3 mM induced a sustained increase in $[Ca^{2+}]i$ in wild-type, P2X7-CT2 Tg, and anti-P2X7-scFv Tg BMDMs (Supplementary Figure S5B). The patterns of change in $[Ca^{2+}]i$ were comparable among wild-type, P2X7-CT2 Tg, and anti-P2X7-scFv Tg BMDMs upon ATP or BzATP stimulation. These results suggest that P2X7-CT2 or anti-P2X7-scFv expression does not affect the cation channel function of endogenous P2X7 in BMDMs.

Supplementary Tables

Table S1. Specific oligonucleotide primers used to amplify anti-P2X7-scFv.

#1: 5′-CGAATgcggccgcGCCACCATGGAGGTTCAGCTGCAGCAGTCTG-3′
#2: 5′-CAGAACCACCACCCCCTGAGGAGACGGTGACTGAGGTTCC-3′
#3: 5′-GGTGGAGGAGGTTCTGATGTTGTGATGACCCAAACTCCACTC-3′
#4: 5′-CTAGtctagaCCGTTTTATTTCCAGCTTGGTCC-3′
#5: 5′-TCACAACATCAGAACCTCCTCCACCGGATCCTCCACCTCCAGAACCACCACCCCC-3′
#6: 5′-CGTCTCCTCAGGGGGTGGTGGTTCTGGAGGTGGAGGATCCGGTGGAGGAGGTTCT-3′

Lowercase letters indicate restriction sites for *Not* I (gcggccgc) and *Xba* I (tctaga).

Table S2. Specific oligonucleotide primers used for PCR amplification.

GenBank number	primers
**BC117057.1**	TNF-α-Forward; 5′-CTGTAGCCCACGTCGTAGC-3′
	TNF-α-Reverse; 5′-TTGAGATCCATGCCGTTG-3′
**M15131.1**	IL-1β-Forward; 5′-TGTAATGAAAGACGGCACACC-3′
	IL-1β-Reverse; 5′-TCTTCTTTGGGTATTGCTTGG-3′

**M24221.1**	IL-6-Forward; 5′-TCTAATTCATATCTTCAACCAAGAGG-3′
	IL-6-Reverse; 5′-TGGTCCTTAGCCACTCCTTC-3′
**J00423.1**	HPRT-Forward; 5′-TGATAGATCCATTCCTATGACTGTAGA-3′
	HPRT-Reverse; 3′-AAGACATTCTTTCCAGTTAAAGTTGAG-3′

GenBank numbers indicate the genes used to design the primers.

References

1. Takenouchi T, Suzuki S, Shinkai H, Tsukimoto M, Sato M, Uenishi H, et al. Extracellular ATP does not induce P2X7 receptor-dependent responses in cultured renal- and liver-derived swine macrophages. *Results Immunol* (2014) 4:62-67. doi: 10.1016/j.rinim.2014.07.002.
2. Takenouchi T, Ogihara K, Sato M, Kitani H. Inhibitory effects of U73122 and U73343 on Ca^{2+} influx and pore formation induced by the activation of P2X7 nucleotide receptors in mouse microglial cell line. *Biochim Biophys Acta* (2005) 1726, 177-186. doi: 10.1016/j.bbagen.2005.08.001.
